# An artificial intelligence real-time rare sperm detection system for intraoperative microsurgical testicular sperm extraction

**DOI:** 10.1093/hropen/hoag062

**Published:** 2026-07-13

**Authors:** Sha Han, Shuai Xu, Haojun Hu, Zijue Zhu, Furong Bai, Cunzhong Deng, Yuhua Huang, Chenwang Zhang, Yifan Sun, Haowei Bai, Chencheng Yao, Fujun Zhao, Zheng Li, Dengyang Zhao, Peng Li

**Affiliations:** Department of Andrology, The Center for Men’s Health, Urologic Medical Center, Shanghai General Hospital, Shanghai Jiao Tong University School of Medicine, Shanghai, China; Department of Andrology, The Center for Men’s Health, Urologic Medical Center, Shanghai General Hospital, Shanghai Jiao Tong University School of Medicine, Shanghai, China; Department of Artificial Intelligence, College of Information Engineering, China Jiliang University, Hangzhou, China; Department of Andrology, The Center for Men’s Health, Urologic Medical Center, Shanghai General Hospital, Shanghai Jiao Tong University School of Medicine, Shanghai, China; Department of Andrology, The Center for Men’s Health, Urologic Medical Center, Shanghai General Hospital, Shanghai Jiao Tong University School of Medicine, Shanghai, China; Department of Andrology, The Center for Men’s Health, Urologic Medical Center, Shanghai General Hospital, Shanghai Jiao Tong University School of Medicine, Shanghai, China; Department of Andrology, The Center for Men’s Health, Urologic Medical Center, Shanghai General Hospital, Shanghai Jiao Tong University School of Medicine, Shanghai, China; Department of Andrology, The Center for Men’s Health, Urologic Medical Center, Shanghai General Hospital, Shanghai Jiao Tong University School of Medicine, Shanghai, China; Department of Andrology, The Center for Men’s Health, Urologic Medical Center, Shanghai General Hospital, Shanghai Jiao Tong University School of Medicine, Shanghai, China; Department of Andrology, The Center for Men’s Health, Urologic Medical Center, Shanghai General Hospital, Shanghai Jiao Tong University School of Medicine, Shanghai, China; Department of Andrology, The Center for Men’s Health, Urologic Medical Center, Shanghai General Hospital, Shanghai Jiao Tong University School of Medicine, Shanghai, China; Department of Andrology, The Center for Men’s Health, Urologic Medical Center, Shanghai General Hospital, Shanghai Jiao Tong University School of Medicine, Shanghai, China; Department of Andrology, The Center for Men’s Health, Urologic Medical Center, Shanghai General Hospital, Shanghai Jiao Tong University School of Medicine, Shanghai, China; Department of Artificial Intelligence, College of Information Engineering, China Jiliang University, Hangzhou, China; Department of Andrology, The Center for Men’s Health, Urologic Medical Center, Shanghai General Hospital, Shanghai Jiao Tong University School of Medicine, Shanghai, China

**Keywords:** rare sperm, YOLO system, non-obstructive azoospermia, microsurgical testicular sperm extraction, real-time detection, artificial intelligence

## Abstract

**STUDY QUESTION:**

How can rare spermatozoa be identified efficiently during microsurgical testicular sperm extraction (micro-TESE)?

**SUMMARY ANSWER:**

An artificial intelligence (AI)-assisted system was developed to flag candidate rare spermatozoa in real time during micro-TESE, and it may support embryologists as a decision-support tool.

**WHAT IS KNOWN ALREADY:**

Patients with non-obstructive azoospermia (NOA) can obtain sperm for procreation through micro-TESE. During this procedure, sperm retrieval primarily relies on embryologists or laboratory technicians visually searching for sperm under a microscope, which is not only laborious and inherently subjective but also susceptible to errors. Although AI technology has been applied to identify trace amounts of sperm, existing models lack sufficient efficiency and true real-time performance.

**STUDY DESIGN, SIZE, DURATION:**

This study included model development followed by a single-centre clinical evaluation. An improved YOLO (You Only Look Once)-based rare sperm detection model, termed YOLOv11-RSD, was developed using microscopy data from 1165 surgical patients, comprising 1932 image samples containing a total of 5032 annotated sperm objects with confirmed identification. Clinical evaluation was performed between May 2024 and July 2025. Performance was assessed across confidence thresholds in obstructive azoospermia (OA) patients with normal spermatogenesis, and the system was then applied during micro-TESE in NOA patients and compared with routine embryologist assessment.

**PARTICIPANTS/MATERIALS, SETTING, METHODS:**

The model was developed using testicular sperm microscopy images collected at a single hospital. Real-time clinical feasibility was evaluated in 10 OA cases and 30 NOA cases. Embryologist assessment was used as the reference standard, and performance was assessed using PPV, sensitivity, *F*1-score, and 95% confidence intervals. Discordant AI-assisted detections were reviewed by embryologists in real time.

**MAIN RESULTS AND THE ROLE OF CHANCE:**

YOLOv11-RSD achieved real-time detection of candidate spermatozoa in microscopy images with high sensitivity and acceptable PPV under the selected operating threshold. Compared with baseline YOLOv11, YOLOv11-RSD showed improved overall detection performance across representative evaluation settings. In OA cases, the system achieved high sensitivity for sperm detection, reaching up to 96.7% across evaluated thresholds. During micro-TESE in NOA patients, at a confidence threshold of 0.50, positive predictive value (PPV), sensitivity, and *F*1-score were 80.58%, 96.11%, and 87.66%, respectively. The system highlighted candidate spermatozoa that were not identified during the initial manual assessment in six NOA cases, including two cases initially classified as sperm-negative; these findings were confirmed upon immediate re-review. Follow-up reproductive outcomes were available for six cases in which AI-assisted detection contributed to the search-and-confirmation workflow: embryo cleavage was achieved in all six cases, and three cases ultimately resulted in live births. Notably, among the two cases initially classified as sperm-negative, one case resulted in a singleton live birth.

**LARGE SCALE DATA:**

N/A

**LIMITATIONS, REASONS FOR CAUTION:**

This was a single-centre clinical evaluation with a limited clinical cohort. Although model inference was rapid, procedure-level efficiency was constrained by image acquisition and scanning logistics, and no definitive reduction in total procedure time was demonstrated. External multi-centre validation is required.

**WIDER IMPLICATIONS OF THE FINDINGS:**

AI-assisted sperm detection may support embryologists during micro-TESE by flagging candidate rare spermatozoa for rapid review. Further prospective multi-centre validation is required to determine whether this approach improves procedure-level efficiency or clinical outcomes.

**FUNDING:**

This work was supported by grants from National Natural Science Foundation of China (82301794), Shanghai Science and Technology Innovation Action Plan (24Y12800702), Natural Science Foundation of Shanghai (25ZR1401300), National Key Research and Development Program of China (2022YFC270300), China Jiliang University Research Grant (No. H251120), and Shanghai General Hospital Basic and Clinical Collaborative Research Program (JC202612).

**DISCLOSURES:**

The authors declare no competing interests.

WHAT DOES THIS MEAN FOR PATIENTS? Men with non-obstructive azoospermia have no sperm visible in the ejaculate because their testicles produce very few or no sperm. Microsurgical testicular sperm extraction might be performed for sperm retrieval, during which very small pieces of testicular tissue are examined under a microscope to look for rare sperm that may be used for assisted reproductive technology. However, finding sperm during this procedure is very difficult. Sperm may be extremely rare, and the microscope slides also contain many other cells and tissue fragments. At present, trained laboratory specialists need to search the slides by eye, which can take time and could be affected by fatigue.

In this study, we developed an artificial intelligence system designed to help real-time sperm detection during micro-TESE. It does not replace the laboratory specialist and does not collect sperm for treatment. Instead, it acts as a support tool: any possible sperm highlighted by the system still needs to be checked and confirmed by trained staff.

In this single-centre study, the system was able to highlight possible sperm during the operation. In some cases, it pointed out sperm-like cells that were not seen during the first manual check but were confirmed after immediate review. Some of the sperm retrieved from these cases later contributed to embryo development and live births. However, a larger multi-centre study is needed to confirm the efficiency of this novel AI system.

## Introduction

Approximately 15% of infertile patients suffer from azoospermia (Eisenberg *et al.*, 2013). For men with non-obstructive azoospermia (NOA), sperm retrieval usually requires microsurgical testicular sperm extraction (micro-TESE), typically followed by intracytoplasmic sperm injection (ICSI) (Deruyver *et al.*, 2014; Bernie *et al.*, 2015). Although micro-TESE is considered the gold-standard surgical approach for NOA, its sperm retrieval rate remains only about 45%, with a live birth rate of approximately 13% (Vloeberghs *et al.*, 2015). Intraoperatively, testicular tissue is mechanically processed into cell suspensions, which are then examined manually under the microscope in search of rare spermatozoa. This process is labour-intensive, time-consuming, and highly dependent on operator experience. Because only part of the specimen can be reviewed and spermatozoa may be extremely scarce, even experienced embryologists may fail to identify viable sperm (Lee *et al.*, 2022). Consequently, outcomes for NOA are constrained as much by human cognition and endurance as by surgical technique.

Automated sperm detection in microscopy images remains technically challenging. In the micro-TESE setting, missed detections may occur in crowded or complex fields, while cellular debris and heterogeneous background structures can increase false-positive detections (Jamalirad *et al.*, 2025). Attempts have been made to automate sperm identification via microscopy. In contrast to detection-based Multi-Object Tracking (MOT) algorithms, conventional tracking approaches demonstrate significant constraints in environmental adaptability, target recognition and categorization, performance in complex scenarios, and long-term tracking consistency. The enhanced YOLOv8 small target detection algorithm (SpermYOLOv8-E) has been used in semen analysis for sperm detection and tracking (Sato *et al.*, 2022; Zhang *et al.*, 2024). However, these approaches have not been translated to micro-TESE, where the visual environment differs substantially from ejaculate microscopy. Micro-TESE images frequently contain thick tissue layers, cohesive cell clusters with adherent fragments, and rich cellular heterogeneity (e.g., Sertoli cell, Leydig cell, germ cells, erythrocytes, stromal debris). In addition, irregular illumination, shallow depth of field, dense overlap, and variable motion are common. Together, these factors increase both false negatives and false positives, limiting the performance of generic detection or tracking pipelines. Although deep learning has been explored for detecting rare sperm in simulated or contrived semen samples and clinically collected azoospermic samples (Naik *et al.*, 2024; Suryawanshi *et al.*, 2025), these studies have generally relied on semen or freshly processed tissue samples and have not demonstrated practical applicability during live micro-TESE procedures. There remains a clear unmet need in the andrology laboratory for a system that can support rapid and accurate identification of rare spermatozoa during surgery, with the potential to improve workflow efficiency and assist sperm retrieval in challenging NOA cases.

Since its introduction in 2016, the YOLO framework has evolved through multiple generations, achieving significant improvements in both detection speed and accuracy, and becoming one of the most widely used architectures for real-time object detection tasks (Redmon *et al.*, 2016). YOLOv3 introduced multi-scale prediction to enhance small-object detection performance (Redmon and Farhadi, 2018), and later YOLO-family developments further enhanced feature fusion, robustness and computational efficiency for small-target and medical-image applications (Diwan *et al.*, 2023; Wang *et al.*, 2023; Topuz *et al.*, 2024). These advances have enabled YOLO-based models to perform effectively in biomedical imaging, including cell counting, histopathological image analysis, and sperm detection under microscopic conditions (Dobrovolny *et al.*, 2023; Sazak and Kotan, 2024).

Automation to identify sperm in the andrology laboratory could allow more efficient and more effective identification of rare sperm, which would be a substantial advancement in the field of male infertility (Staine *et al.*, 2022). To improve sperm retrieval support in patients with NOA, we designed an improved YOLOv11-based architecture optimized for the micro-TESE environment for accurate real-time intraoperative rare sperm detection. This system enables real-time intraoperative detection, demonstrates high detection accuracy, and may support real-time intraoperative candidate sperm detection within the existing micro-TESE workflow.

## Materials and methods

### Ethics statement

Ethical approval was obtained for this study. The study was designed by the investigators and conducted in accordance with the Declaration of Helsinki and Good Clinical Practice guidelines. The study was approved by the Ethics Committee of Shanghai General Hospital (approval no. 2023-108). All participants provided written informed consent before enrolment.

### Sample preparation

Micro-TESE testicular samples were obtained from patients diagnosed with NOA. NOA was defined as azoospermia on two separate semen analyses after centrifugation, with obstructive azoospermia excluded by clinical evaluation and scrotal ultrasonography. Additional supportive findings included elevated serum follicle-stimulating hormone levels and reduced testicular volume on examination.

### YOLO enhancement

**Figure 1.**
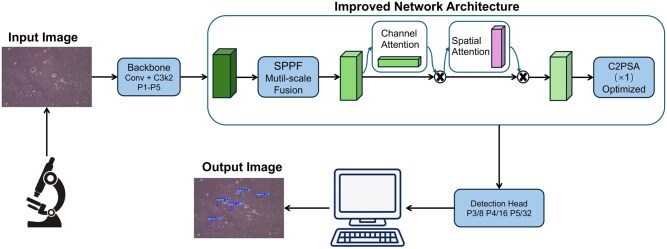
**YOLOv11-RSD model architecture**. YOLOv11-RSD (You Only Look Once version 11-rare sperm detection) employs an end-to-end detection pipeline. Input sperm microscopy images first undergo hierarchical feature extraction through the Backbone network, including multi-layer convolutions, SPPF (Spatial Pyramid Pooling-Fast) multi-scale feature fusion module, CBAM (Convolutional Block Attention Module) attention module, and C2PSA (C2 Position-Sensitive Attention) global context modeling module. Subsequently, the Neck feature fusion layer completes cross-scale feature interaction and propagation. Finally, three detection heads (P3, P4, P5) perform object detection at different scales, outputting detection results with annotated bounding boxes. The overall architecture forms a complete pipeline of ‘feature extraction→attention refinement→global modeling→multi-scale detection’.

To improve real-time detection of extremely rare spermatozoa, we developed an optimized YOLO-based model, YOLOv11-RSD (Fig. 1). The optimization was designed to strengthen small-target recognition, reduce potential missed detections in complex microscopic backgrounds, and maintain intraoperative real-time speed.

First, we inserted a Convolutional Block Attention Module (CBAM) to enhance sperm-relevant features while suppressing background structures that can mimic sperm-like signals (e.g. tissue fragments and debris). CBAM was placed between Spatial Pyramid Pooling-Fast (SPPF) (multi-scale feature aggregation) and C2PSA (C2 Position-Sensitive Attention) (global feature integration), so that information from different receptive fields is first fused, then selectively emphasized through spatial/channel attention, and finally integrated for detection—an arrangement intended to improve sensitivity to rare spermatozoa, particularly in dense or visually cluttered fields. Second, to improve efficiency without compromising modeling capacity, we reduced the repetition of the C2PSA block from two to one, lowering computational load and supporting stable real-time inference. Third, we adopted a three-scale detection scheme with three heads (P3/P4/P5) to enable robust multi-scale recognition across heterogeneous specimen conditions. In this hierarchical design, the P3 head primarily targets small spermatozoa in densely clustered regions, the P4 head provides balanced detection under typical fields, and the P5 head supports detection in sparser fields and for relatively larger-scale targets. Overall, these modifications were intended to improve robustness to variation in sperm size, contrast, and sample density, while providing a practical balance between accuracy and speed for intraoperative deployment.

### Evaluation of YOLOv11-RSD

The baseline YOLOv11 model and the enhanced YOLOv11-RSD model were compared using a dataset of 1932 intraoperative testicular sperm microscopy images from 1165 patients. Image annotation was performed independently by three embryologists, each with more than 5 years of experience in sperm identification. Annotations were generated according to routine laboratory criteria for sperm identification, and discrepancies were resolved by consensus review to generate the final reference annotation set. A formal quantitative interobserver agreement metric, such as kappa, was not calculated in the original annotation workflow, and this has been acknowledged as a limitation. Model performance was assessed on the complete evaluation dataset under six combinations of intersection-over-union (IoU) and confidence thresholds: (a) IoU = 0.3, confidence = 0.3; (b) IoU = 0.5, confidence = 0.3; (c) IoU = 0.4, confidence = 0.4; (d) IoU = 0.6, confidence = 0.4; (e) IoU = 0.5, confidence = 0.5; and (f) IoU = 0.7, confidence = 0.5. These settings represented evaluation criteria ranging from relatively lenient to more stringent. Four performance metrics were analysed: mean average precision across IoU thresholds from 0.50 to 0.95 (mAP_50–95_), precision, recall, and *F*1-score. For clinical interpretation, precision and recall are also referred to as positive predictive value (PPV) and sensitivity, respectively.

### Real-time detection of sperm in testicular suspensions from obstructive azoospermia patients

Testicular tissues were obtained from 10 patients with obstructive azoospermia (OA) at Shanghai General Hospital. All patients provided written informed consent, and subsequent histological analysis confirmed normal spermatogenesis.

A scientific Complementary Metal-Oxide-Semiconductor (sCMOS) camera (MS60, Mshot, Guangzhou, China) was connected to the microscope to display the microscopic field in real time on a computer monitor. The live video feed was processed by YOLOv11-RSD, which continuously analyzed the images to detect sperm and record the procedure (Supplementary Fig. S1). Throughout the process, the number of sperm identified by both the embryologist and YOLOv11-RSD system was recorded frame by frame.

Following the real-time analysis, video recordings were reviewed frame-by-frame. Each sperm candidate identified by the YOLOv11-RSD system was cross-referenced with the embryologist’s annotations to determine true positives. False negatives were also recorded by reviewing all frames for any sperm missed by the YOLOv11-RSD system.

### Real-time sperm detection during micro-TESE using YOLOv11-RSD

All micro-TESE procedures were conducted under general or spinal anesthesia as per established guidelines (Schlegel and Li, 1998), with minor modifications. An incision was made in the testis along the scrotal midline. Subsequently, an incision was made in the tunica albuginea under an operating microscope (Vario700, Carl Zeiss Shanghai Co., Ltd., Shanghai, China) to expose the testicular parenchyma while preserving the blood supply. Intraoperatively, a “wet prep” of the suspension was examined under phase-contrast microscopy at ×200 power. A sCMOS camera (MS60, Mshot, Guangzhou, China) remained connected to the microscope, transmitting the microscopic field in real time to a computer screen for analysis by YOLOv11-RSD. The wet prep was manually scanned by an embryologist, following a routine clinical search for sperm. Throughout the process, YOLOv11-RSD continuously analyzed the video feed, identifying sperm and recording all detections in real time (Supplementary Fig. S1). The total time required for the complete manual scanning of the wet prep was recorded and the number of sperm detected by YOLOv11-RSD was tallied and subsequently compared against the embryologist’s findings during the scan. In cases with negative sperm counts, after sperm-like objects were flagged by YOLOv11-RSD, we immediately consulted a second embryologist for confirmation. We also recorded the subsequent biological verification results for the patients. Simultaneously, we strictly adhered to microscopic sperm retrieval standards, examining the entire wet prep, with both experts verifying the presence of sperm. Furthermore, our system is based on real-time, synchronous display of microscopic images, consistent with the field of view observed by the embryologist.

### Clinical workflow for discordant AI and embryologist assessments

In the current workflow, YOLOv11-RSD was used as a decision-support tool running in parallel with embryologist assessment. When the AI system flagged a sperm-like object that had not been identified during the initial manual assessment, the field was immediately paused or revisited, and the candidate object was reviewed by the primary embryologist. If the finding remained uncertain or if the procedure had initially been considered sperm-negative, a second embryologist was consulted for real-time confirmation. Only spermatozoa confirmed by embryologist review were considered clinically usable. When the embryologist identified spermatozoa that were not flagged by the AI system, the embryologist’s assessment took precedence, and the event was recorded as a model miss for quality control. All discordant events were logged for subsequent audit and model refinement.

### Statistical analyses

For diagnostic performance of the YOLOv11-RSD system, true positives (TP), false positives (FP), false negatives (FN), and true negatives (TN) were obtained by comparing model outputs with embryologist annotations. Positive predictive value (PPV, precision) was calculated as TP/(TP + FP); sensitivity (recall) was calculated as TP/(TP + FN); and *F*1-score was calculated as 2 × PPV × sensitivity/(PPV + sensitivity).

Performance metrics are presented with 95% confidence intervals. The 95% confidence intervals (CIs) for PPV (precision) and sensitivity (recall) were calculated using Wilson score intervals. The 95% CI for *F*1-score was obtained by bootstrap resampling (*B* = 20 000) with percentile intervals. Statistical analyses, including Wilson score confidence intervals and bootstrap resampling, were performed using Python 3.10 (Python Software Foundation, Wilmington, DE, USA).

## Results

### YOLOv11-RSD system evaluation

**Figure 2.**
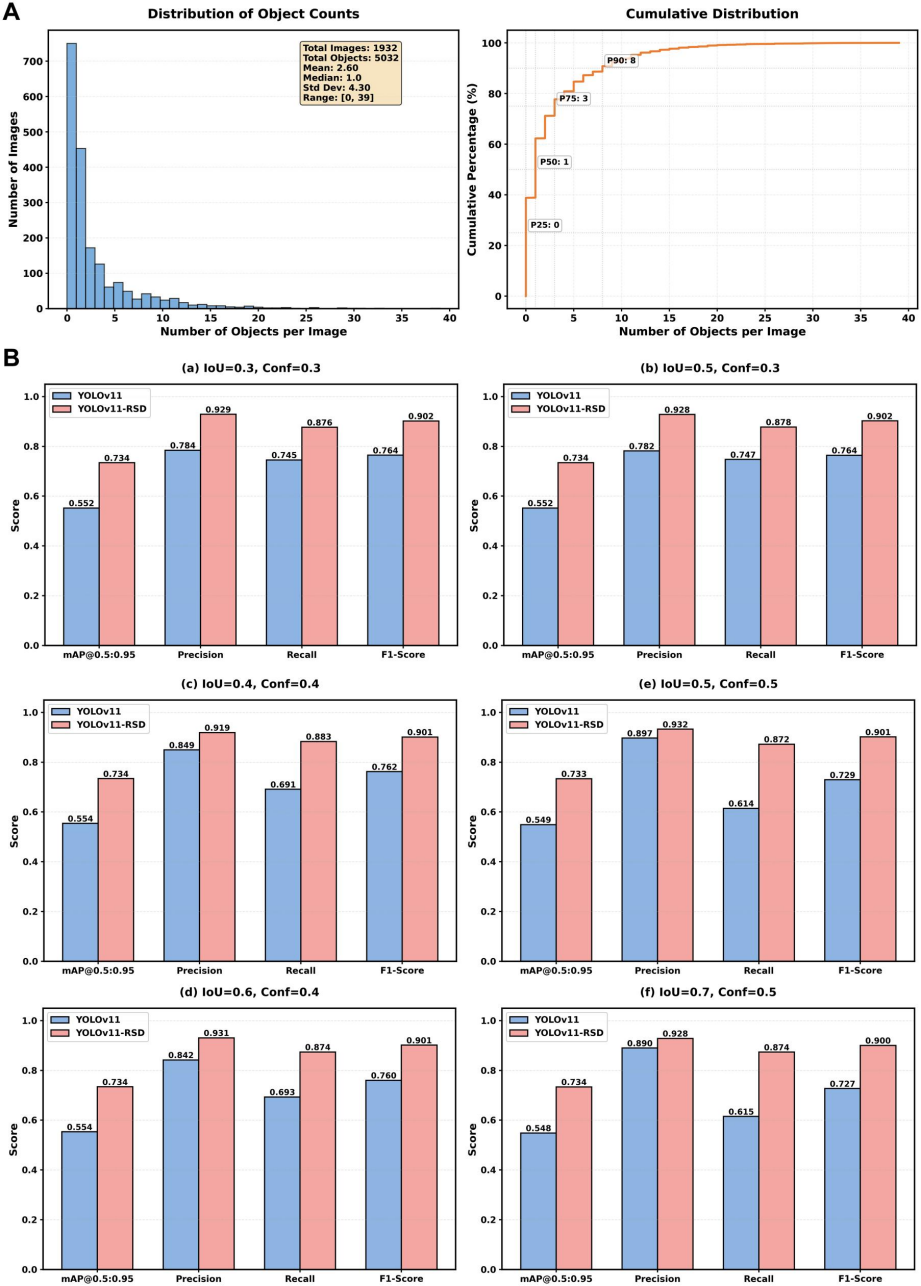
**Effectiveness of the proposed improvements**. (**A**) Distribution of sperm object counts per image and cumulative distribution function. (**B**) Performance comparison between baseline YOLOv11 and YOLOv11-RSD across representative IoU-confidence thresholds. YOLOv11-RSD consistently outperformed baseline YOLOv11 under lenient, standard, and stringent evaluation settings, with higher mAP_50–95_, PPV, sensitivity, and *F*1-score. Under the standard setting (IoU = 0.5, confidence = 0.5), the improved model showed fewer false negatives and false positives, together with a higher true positive rate, supporting the robustness of the proposed enhancements. IoU, intersection over union; mAP50–95, mean average precision across IoU thresholds from 0.50 to 0.95; precision: PPV (positive predictive value); recall: sensitivity.

To evaluate the effectiveness of the proposed improvements, we compared baseline YOLOv11 with YOLOv11-RSD across multiple parameter settings. The dataset comprised 1932 images with 5032 annotated objects from 1165 patients, randomly split into training, validation, and test sets (Fig. 2A). Model performance was assessed under representative IoU-confidence thresholds ranging from lenient to stringent, using mAP_50–95_, PPV, sensitivity, and *F*1-score. Under a lenient setting (IoU = 0.5, Conf = 0.3), YOLOv11-RSD showed higher mAP_50–95_ (0.734 vs. 0.552), PPV (0.928 vs. 0.782), and sensitivity (0.878 vs. 0.747). At the standard setting (IoU = 0.5, Conf = 0.5), improvements were maintained (mAP_50–95_: 0.733 vs. 0.549; sensitivity: 0.872 vs. 0.614). Paired confusion matrices demonstrated reduced false negatives (38.6% to 12.8%) and false positives (10.3% to 6.8%), with increased true positives (61.4% to 87.2%). Similar gains under stricter criteria (IoU = 0.7, Conf = 0.5) confirm the robustness of the model (Fig. 2B; Supplementary Fig. S2).

**Figure 3.**
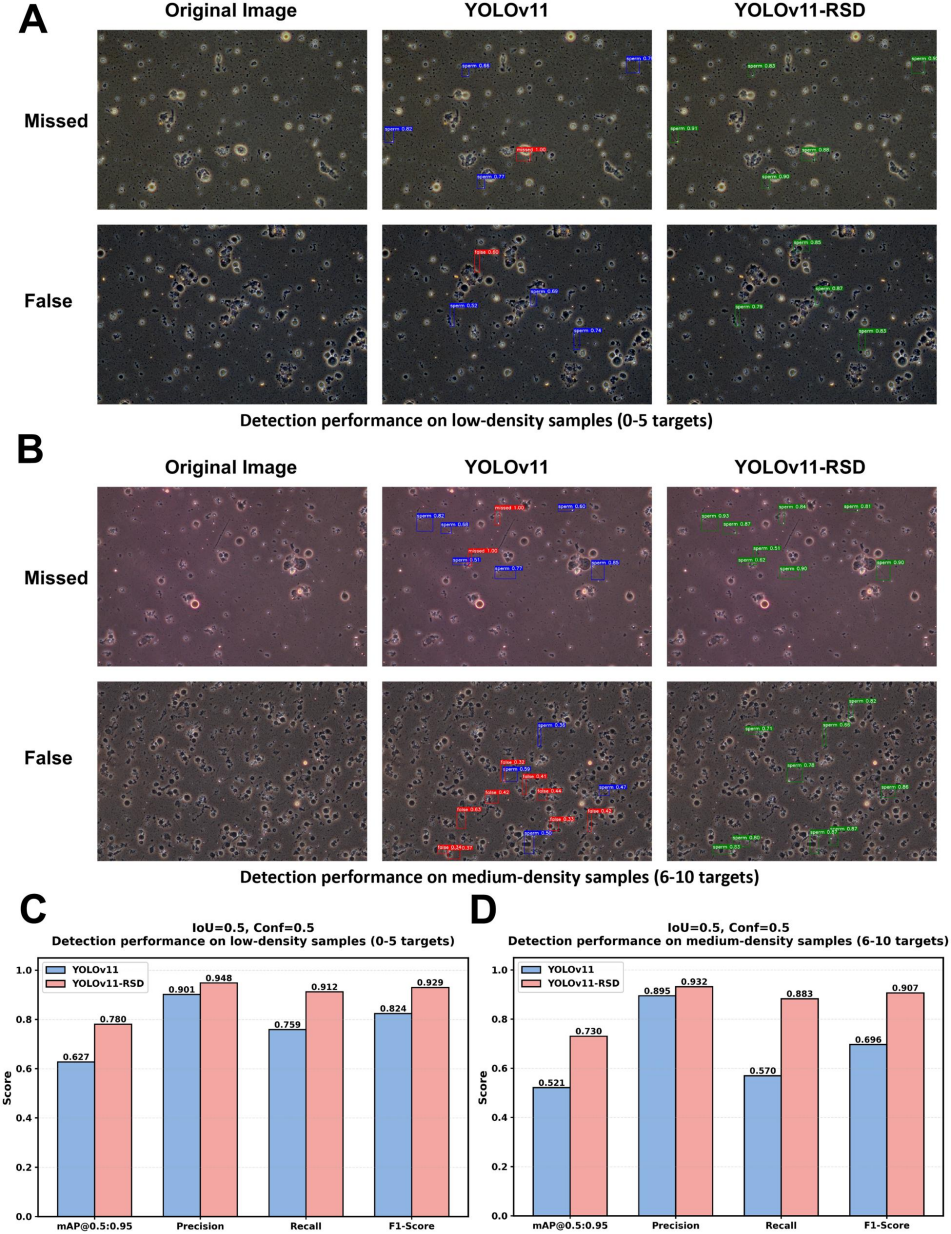
**Representative detection outcomes across different sample densities**. (**A**, **C**) Low-density samples (0–5 targets). (**B**, **D**) Medium-density samples (6–10 targets). Representative examples and quantitative analyses show that YOLOv11-RSD outperformed baseline YOLOv11 across different sample densities. In both low- and medium-density samples, the improved model reduced missed detections and false positives (shown in red), particularly in crowded regions. Sensitivity increased from 75.9% to 91.2% in low-density samples and from 57.0% to 88.3% in medium-density samples. YOLOv11-RSD, You Only Look Once version 11-rare sperm detection; precision: PPV (positive predictive value); recall: sensitivity.

Representative cases across varying sample densities illustrate the practical performance of the system. In low-density samples (0–5 targets), the baseline YOLOv11 missed sperm and produced occasional false positives, whereas YOLOv11-RSD correctly identified all targets while suppressing background noise (Fig. 3A). In medium-density samples (6–10 targets), the baseline model missed targets in crowded regions and generated multiple false positives, while YOLOv11-RSD achieved accurate detection with markedly fewer errors (Fig. 3B). Quantitative analysis (Fig. 3C and D) confirmed these findings. In low-density samples, sensitivity improved from 75.9% to 91.2%, while in medium-density samples it increased from 57.0% to 88.3%, demonstrating a substantial gain under more challenging conditions. Together, these results highlight the model’s ability to improve detection accuracy while reducing false positives across different sample densities.

**Figure 4.**
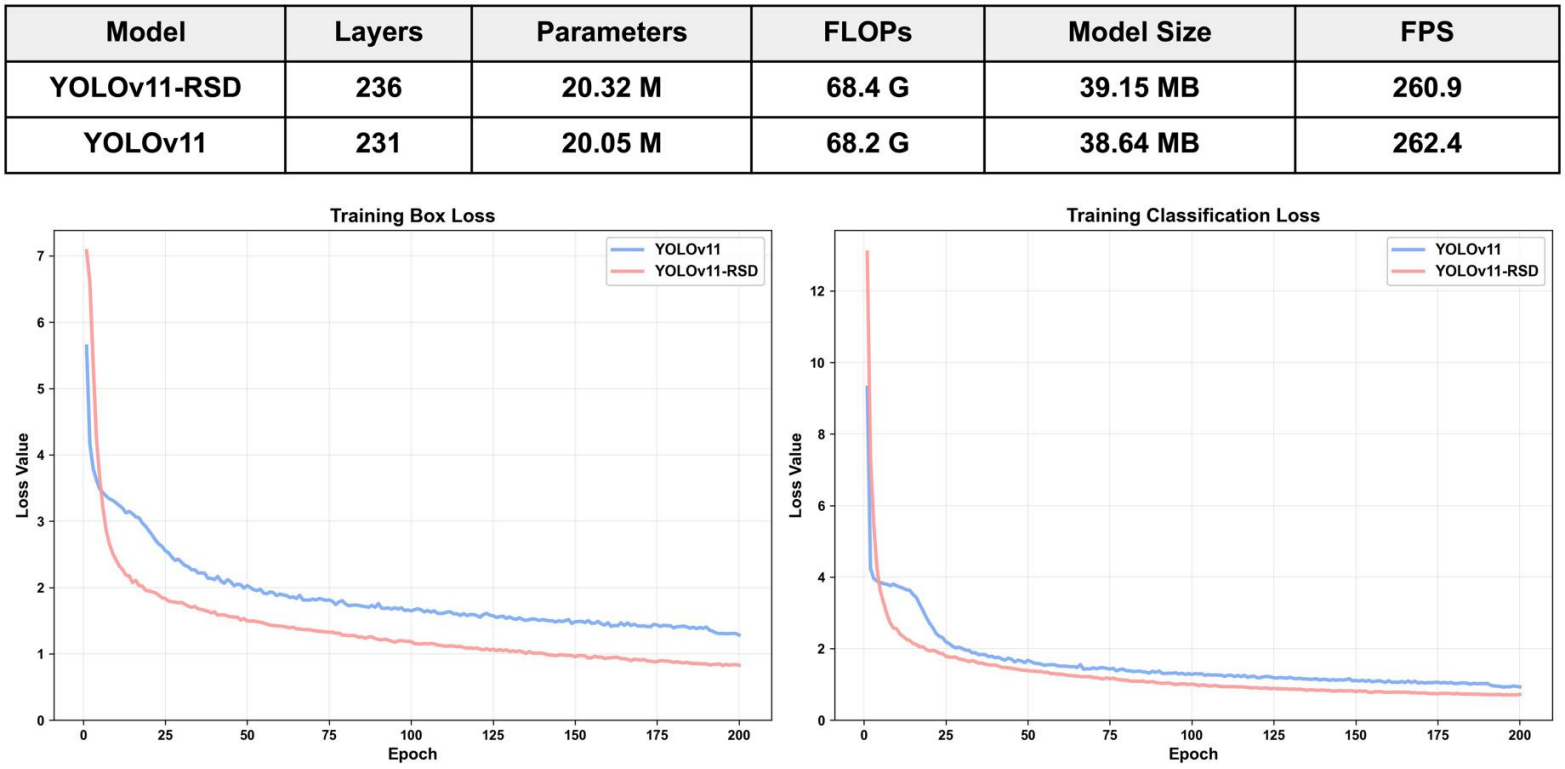
**Comparison of model complexity between the YOLOv11-RSD model and the YOLOv11**. (**A**) Computational overhead comparison. (**B**) Analysis of the box and classification loss curves. YOLOv11-RSD introduced only minimal increases in model complexity, with little change in inference speed. During training, the improved model showed faster convergence and lower final loss values, supporting its practical feasibility for clinical application. CBAM, Convolutional Block Attention Module; FLOPs, floating-point operations; FPS, frames per second; YOLOv11-RSD, You Only Look Once version 11-rare sperm detection.

Model complexity analysis showed that incorporating CBAM introduced minimal overhead, with only slight increases in layers (231 to 236), parameters (20.05 to 20.32 M), floating-point operations (FLOPs; 68.2 to 68.4 G), and model size (38.64 to 39.15 MB). Inference speed remained essentially unchanged, with YOLOv11-RSD achieving 260.9 frames per second (FPS) versus 262.4 FPS for YOLOv11 at 640 × 640 resolution (Fig. 4A).

During training, YOLOv11-RSD demonstrated faster convergence and lower final loss values, with box loss decreasing from 1.283 to 0.829 and classification loss decreasing from 0.929 to 0.720 (Fig. 4B). These results indicate improved learning efficiency and feature representation, while maintaining practical feasibility for real-world clinical deployment.

### Real-time sperm detection in testicular biopsy samples with normal spermatogenesis using the YOLOv11-RSD system

**Figure 5.**
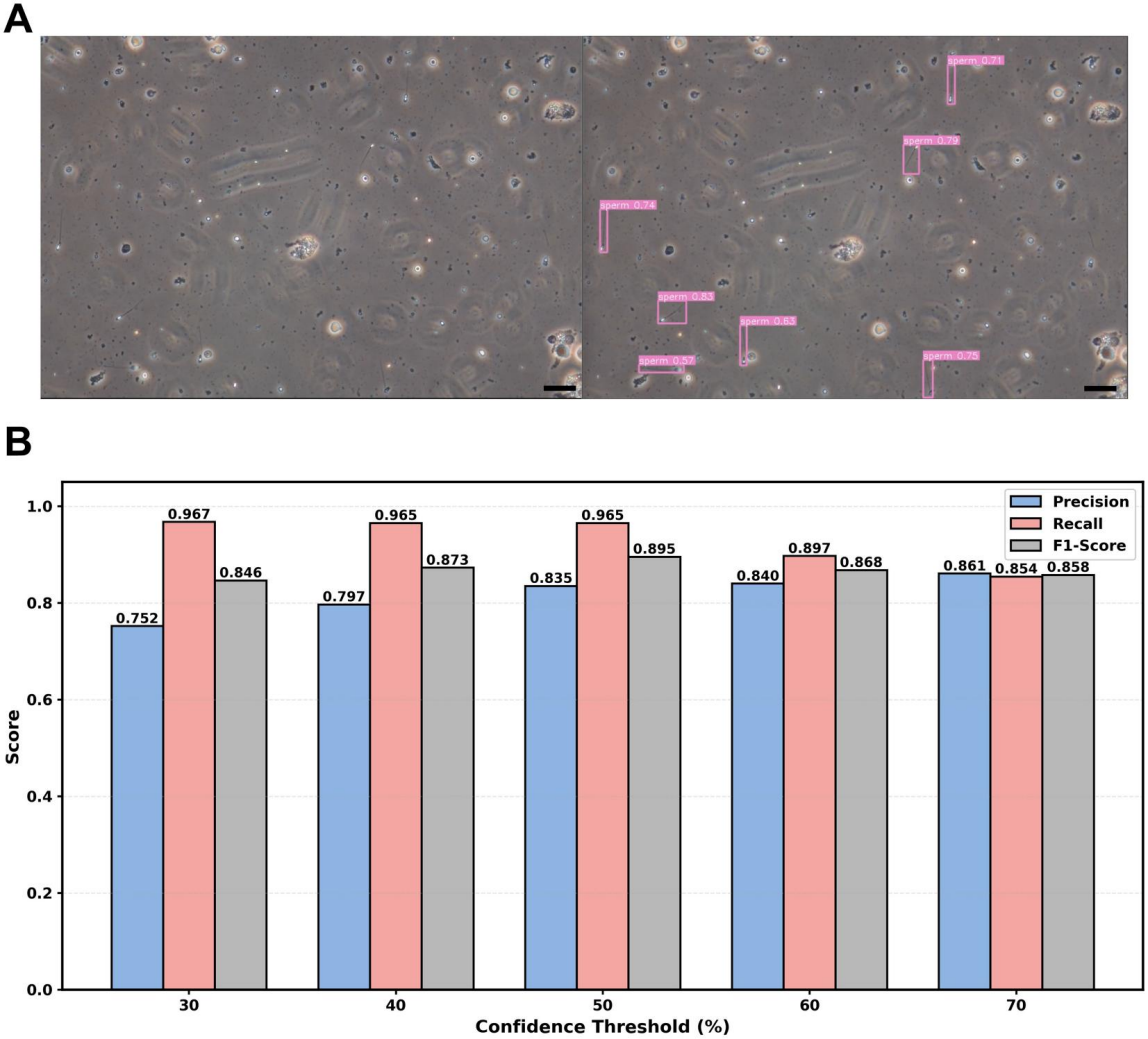
**Representative real-time YOLOv11-RSD-assisted sperm detection in patients with obstructive azoospermia**. (**A**) Intraoperative microscope real-time imaging (Left) and real-time YOLO detection with 50% confidence threshold (Right) (**B**) PPV (positive predictive value, Precision), sensitivity (Recall), and F1-scores for detecting sperm in testis tissue using different confidence thresholds. The scale bar represents 50 μm. YOLOv11-RSD, You Only Look Once version 11-rare sperm detection; OA, obstructive azoospermia; PPV, positive predictive value; sensitivity, recall.

Among 10 patients with obstructive azoospermia (OA) and normal spermatogenesis, real-time sperm detection was performed using the YOLOv11-RSD system at confidence thresholds of 30%, 40%, 50%, 60%, and 70%, with 50 microscopic fields examined at each threshold (Fig. 5A and B). Detection errors included both false positives and false negatives (Supplementary Fig. S3).

Using embryologist assessment as the reference standard, we calculated PPV, sensitivity, *F*1-score, and corresponding 95% confidence intervals (CIs) for each threshold (Supplementary Table S1). At confidence thresholds of 30%, 40%, 50%, 60%, and 70%, PPV was 75.2%, 79.7%, 83.5%, 84.0%, and 86.1%, respectively, while sensitivity was 96.7%, 96.5%, 96.5%, 89.7%, and 85.4%. The corresponding *F*1-scores were 84.6%, 87.3%, 89.5%, 86.8%, and 85.8%.

The operating threshold was selected based on a predefined criterion of maximizing *F*1-score while maintaining high sensitivity. Across all thresholds, the optimal performance was observed at a confidence threshold of 50%.

### Real-time rare sperm detection during micro-TESE using the YOLOv11-RSD system

**Figure 6.**
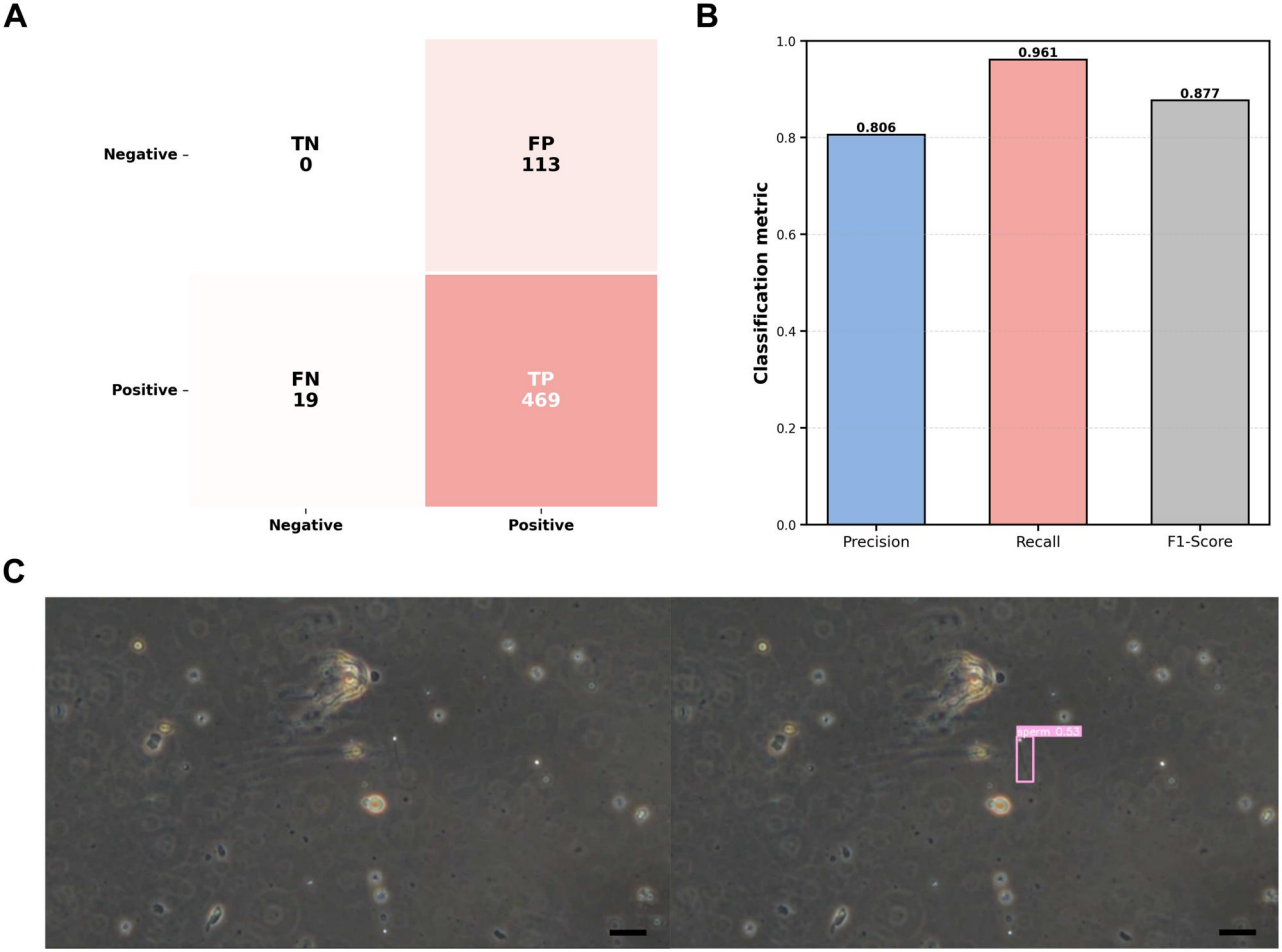
**Representative real-time YOLOv11-RSD-assisted sperm detection in patients with non-obstructive azoospermia**. (**A**) Confusion matrices of YOLOv11-RSD. (**B**) PPV (positive predictive value, Precision), sensitivity (Recall), and *F*1-scores for detecting sperm in testis tissue during micro-TESE. (**C**) Representative images showing that YOLOv11-RSD detected rare sperm (right), whereas the embryologist initially considered the procedure sperm-negative (left). The scale bar represents 50 μm. YOLOv11-RSD, You Only Look Once version 11-rare sperm detection; NOA, non-obstructive azoospermia; micro-TESE, microsurgical testicular sperm extraction; PPV, positive predictive value; sensitivity, recall; TP, true positive; FP, false positive; FN, false negative; TN, true negative.

Based on the YOLOv11-RSD tests in OA patients, which showed that the 50% confidence level provided the highest *F*1-score and exceptionally high sensitivity, a 50% confidence threshold was selected for sperm detection during micro-TESE procedures in NOA patients. Among 30 NOA patients, we performed real-time scanning with the YOLOv11-RSD system across diverse etiologies and successfully retrieved sperm in 12 patients, including cases of Klinefelter syndrome, Yq azoospermia factor c deletions, mumps orchitis, and idiopathic NOA (Table 1). During scanning, the sCMOS camera operated at a frame rate of up to 60 frames per second, and the real-time detection response time for each field of view was 8.76 ms. The comparison of model outputs with embryologist identifications showed that, at the 50% confidence level, the PPV, sensitivity, and F1-score were 80.58% (95% CI 0.772–0.836), 96.11% (95% CI 0.940–0.975), and 87.66% (95% CI 0.855–0.897), respectively (Fig. 6A and B). Notably, in six patients, YOLOv11-RSD highlighted candidate spermatozoa that were not identified during the initial manual assessment. More importantly, in two patients, YOLOv11-RSD detected rare sperm, whereas the embryologist initially considered these procedures sperm-negative (Fig. 6C). Follow-up reproductive outcomes were available for six cases in which AI-assisted detection contributed to the search-and-confirmation workflow for sperm retrieval: embryo cleavage was achieved in all six cases, and three cases ultimately resulted in live births. Notably, among the two cases initially classified as sperm-negative, one resulted in a singleton live birth (Table 1).

**Table 1.** Rare sperm detection of wet prep during micro-TESE procedure among NOA patients and clinical reproductive outcomes.

Patient no.	Aetiology	Detection number					Intracytoplasmic sperm injection results			
		Manual	AI	Initial manual non-identification	AI false-positive candidates	Confirmed Manual−/AI+ detections	MII oocyte injected	Normal fertilization (2PN)	Embryo cleaved	Outcome
1	KS	0	5	5	0	0	–	–	–	–
2	Idiopathic	53	59	8	2	0	11	9	9	No pregnancy
3	Idiopathic	1	7	6	0	0	3	1	1	No pregnancy
4	Idiopathic	0	4	4	0	0	–	–	–	–
5	Idiopathic	44	52	7	1	2*	16	13	13	Delivery (single live birth)
6	Idiopathic	0	0	0	0	0	–	–	–	–
7	Idiopathic	0	0	0	0	0	–	–	–	–
8	Idiopathic	0	8	6	0	2*	6	6	6	Delivery (single live birth)
9	Idiopathic	0	0	0	0	0	–	–	–	–
10	Idiopathic	66	72	8	2	0	12	11	9	Pregnancy (miscarriage)
11	Idiopathic	0	4	4	0	0	–	–	–	–
12	Idiopathic	0	0	0	0	0	–	–	–	–
13	KS	0	9	7	0	2*	7	5	2	Pregnancy (miscarriage)
14	Mumps orchitis	77	84	6	2	3*	16	6	5	Pregnancy (miscarriage)
15	Idiopathic	0	0	0	0	0	–	–	–	–
16	Idiopathic	0	0	0	0	0	–	–	–	–
17	KS	51	53	5	3	0	15	15	15	Delivery (single live birth)
18	Idiopathic	0	0	0	0	0	–	–	–	–
19	KS	0	6	6	0	0	–	–	–	–
20	KS	0	0	0	0	0	–	–	–	–
21	AZFc	2	6	5	1	0	3	1	1	Pregnancy (miscarriage)
22	Idiopathic	0	0	0	0	0	–	–	–	–
23	Idiopathic	0	5	5	0	0	–	–	–	–
24	AZFc	61	68	6	3	4*	6	6	5	Delivery (single live birth)
25	Idiopathic	0	0	0	0	0	–	–	–	–
26	Idiopathic	0	0	0	0	0	–	–	–	–
27	KS	78	85	5	1	3*	35	28	28	Pregnancy (miscarriage)
28	Idiopathic	0	5	5	0	0	–	–	–	–
29	KS	39	44	9	4	0	14	13	13	Delivery (single live birth)
30	Idiopathic	0	6	6	0	0	–	–	–	–

micro-TESE: microsurgical testicular sperm extraction; NOA: non-obstructive azoospermia; KS, Klinefelter syndrome; AZFc, azoospermia factor c deletion; PPV, positive predictive value; MII, metaphase II; 2PN, two pronuclei; ICSI, intracytoplasmic sperm injection; AI, artificial intelligence.

Detected sperm counts refer to spermatozoa identified within the analyzed wet-prep microscopy fields during micro-TESE and should not be interpreted as an exhaustive count of all spermatozoa in the retrieved testicular tissue.

AI-assisted detection indicates candidate spermatozoa flagged by YOLOv11-RSD that prompted embryologist re-review. YOLOv11-RSD did not directly isolate or select spermatozoa for ICSI; any clinically usable spermatozoon required embryologist confirmation and manual recovery according to routine laboratory practice.

* Patients with confirmed Manual−/AI+ discordant detections after immediate embryologist re-review.

## Discussion

Identifying rare spermatozoa in testicular biopsy specimens from men with NOA is a demanding task that remains heavily dependent on operator experience, concentration, and endurance. In this study, YOLOv11-RSD improved sperm detection under real intraoperative micro-TESE conditions, reduced operator-dependent variability, and provided synchronous AI-assisted recognition during microscopic screening. These findings suggest that AI may offer practical support in a setting where the rarity of target cells and the complexity of the background make manual detection particularly challenging.

Intraoperatively, a “wet prep” of the suspension was examined under phase-contrast microscopy at ×200 power. We adopted a similar methodological approach; however, the average detection time per sample in our setting was 366.4 s, which was longer than that reported previously (Ostad *et al.*, 1998). The absence of a clear reduction in total procedure time is an important limitation of the present study. Although inference time was only 8.76 ms per frame, the overall screening speed in routine practice remained constrained mainly by hardware- and workflow-related factors, including slide scanning speed, manual stage movement, field-of-view switching, and focusing. Thus, the current bottleneck lies in image acquisition and scanning rather than in algorithmic latency. Because the present system operates in parallel with embryologist confirmation, time reduction was not the primary endpoint at this stage; instead, the main objective was to improve detection accuracy and sensitivity while fitting within the existing clinical workflow. Future gains in efficiency are therefore more likely to depend on improvements in the imaging and scanning pipeline than on further acceleration of the algorithm alone.

Previous deep-learning studies on sperm detection have mainly focused on semen analysis, simulated micro-TESE settings, or clinically collected azoospermic samples, and therefore only partly reflect the challenges of true intraoperative use (Lee *et al.*, 2022; Zhang *et al.*, 2024; Suryawanshi *et al.*, 2025). A major strength of the present study is that YOLOv11-RSD was evaluated directly in the real micro-TESE environment rather than in semen or simulated laboratory conditions. In this setting, testicular spermatozoa typically show weak or minimal motility and are embedded within a far more heterogeneous and cluttered background than ejaculated sperm. Mechanically dissociated testicular tissue contains a complex suspension of Sertoli cells, Leydig cells, germ cells, erythrocytes, tissue fragments, and debris, often under suboptimal illumination and shallow depth of field, making direct translation of existing sperm detection or tracking models difficult. For example, Sato *et al.* combined YOLOv3-based detection with SORT-based tracking, which enabled simultaneous morphological assessment and tracking with high speed, but performance remained influenced by sperm movement, image clarity, and focal depth (Sato *et al.*, 2022). By contrast, YOLOv11-RSD was designed to prioritize clinical robustness and low latency in the dense, noisy microscopic fields typical of micro-TESE. By integrating CBAM-based attention refinement, lightweight global modelling, and multi-scale detection heads, the system maintained reliable performance even in the presence of overlapping spermatozoa and background debris, achieving a practical balance between PPV, sensitivity, robustness, and real-time feasibility for intraoperative sperm detection.

The primary aim of micro-TESE is sperm retrieval in men with NOA; accordingly, sensitivity is a key performance metric in this setting. Because spermatozoa may be extremely scarce, failure to detect even a single sperm could have direct procedural consequences. In our threshold analysis, lowering the confidence threshold produced only minimal gains in sensitivity, but at the cost of a substantial reduction in PPV. By contrast, a confidence threshold of 50% provided the most appropriate balance, achieving the highest *F*1-score while preserving high sensitivity and acceptable PPV. YOLOv11-RSD highlighted candidate spermatozoa that were not identified during the initial manual assessment in six of the 30 NOA cases. Follow-up reproductive outcome data were available for six cases in which AI-assisted detection contributed to the search-and-confirmation workflow: embryo cleavage was achieved in all cases, and three ultimately resulted in live births. Notably, among the two cases initially classified as sperm-negative, one resulted in a singleton live birth. These observations should be interpreted as supportive clinical observations rather than conclusive evidence of clinical superiority. They suggest that AI-assisted detection may help flag candidate spermatozoa for embryologist re-review in selected micro-TESE cases. For cases initially recorded as sperm-negative by the primary embryologist, whenever YOLOv11-RSD flagged a sperm-like object, we immediately requested confirmation by a second embryologist. Both embryologists then re-evaluated the findings and verified the presence of sperm according to standard microscopic sperm retrieval procedures. In addition, we documented downstream reproductive outcomes in cases where AI-assisted detection contributed to the search-and-confirmation workflow. As a result, in its current form, the AI module is intended to serve as a decision-support tool rather than an autonomous system.

This study also has limitations in terms of scale and generalizability. Although annotations were performed by three experienced embryologists and resolved by consensus, a formal quantitative interobserver agreement metric, such as kappa, was not calculated in the original annotation workflow. The clinical evaluation cohort of 10 OA and 30 NOA cases is limited by the single-centre design. Although the model development dataset was substantially larger, comprising 1165 patients and 1932 microscopy images containing 5032 annotated sperm objects, all data were acquired at a single centre using one imaging platform and a standardized workflow. Variability in microscope magnification, optical parameters, image resolution, sample preparation, and laboratory practice may introduce domain shifts that affect model performance in other settings. Importantly, YOLOv11-RSD was used to support visual identification and field prioritization, not to directly isolate spermatozoa for ICSI. Any clinically usable spermatozoon still required embryologist confirmation and manual recovery according to routine laboratory practice. Accordingly, multi-centre external validation, together with calibration and standardization strategies, will be necessary before broader clinical implementation can be considered.

Overall, this study supports the feasibility of integrating AI-assisted sperm detection into the intraoperative micro-TESE workflow. Rather than serving as a purely methodological contribution, YOLOv11-RSD was developed to address a clinically important problem: the time-consuming and operator-dependent identification of extremely rare spermatozoa in men with NOA. While further external validation and workflow optimization are needed, the present findings indicate that AI-assisted detection may provide useful clinical support in this challenging setting.

## Conclusion

Our artificial intelligence detection system demonstrated the ability to identify candidate rare spermatozoa in real time during micro-TESE. By integrating multi-scale fusion, attention refinement, and lightweight global modelling, YOLOv11-RSD enhances the rare sperm detection accuracy and efficiency while reducing operator dependency and variability. The system is a promising decision-support tool to flag candidate fields for embryologist review. Moreover, further prospective multi-centre studies with standardized imaging workflows and procedure-level efficiency endpoints would broaden its use in clinical implementation.

## Supplementary Material

hoag062_Supplementary_Data

## Data Availability

The data underlying this article will be shared on reasonable request to the corresponding author.
