# Supplementary material for: An artificial intelligence real-time rare sperm detection system for intraoperative microsurgical testicular sperm extraction
Source: Hum Reprod Open. 2026 Jul 13;2026(3):hoag062. doi: 10.1093/hropen/hoag062 (PMC13437015; doi:10.1093/hropen/hoag062)
Supplement: Supplementary file 1 [file hoag062_supplementary_data.docx]

**Supplementary Figure S1. YOLOv11-RSD-assisted rare sperm detection system.** A scientific Complementary Metal-Oxide-Semiconductor (sCMOS) camera was connected to the microscope to display wet-prep microscopic fields in real time on a computer monitor. The live video feed was processed by YOLOv11-RSD, which continuously analysed the images to flag candidate spermatozoa and record the procedure. Throughout the process, candidate spermatozoa identified by YOLOv11-RSD were compared with embryologist assessment frame by frame. YOLOv11-RSD, You Only Look Once version 11-rare sperm detection.

**Supplementary Figure S2. Paired confusion matrices under standard evaluation settings with different IoU and confidence thresholds.** IoU, intersection over union; TP, true positive; FP, false positive; FN, false negative; TN, true negative; YOLOv11-RSD, You Only Look Once version 11-rare sperm detection.

**Supplementary Figure S3. Representative false-positive and false-negative detections.** (A) False-positive detection during YOLOv11-RSD-assisted sperm detection (yellow arrow). (B) False-negative detection during YOLOv11-RSD-assisted sperm detection (red arrow). Scale bars, 50 μm. YOLOv11-RSD, You Only Look Once version 11-rare sperm detection.

**Supplementary Table S1 Performance of YOLOv11-RSD at different confidence thresholds with 95% confidence intervals.**

| **Confidence threshold (%)** | **TP**  **(n)** | **FP**  **(n)** | **FN**  **(n)** | **PPV/Precision (95% CI)** | **Sensitivity/Recall (95% CI)** | **F1(95%CI)** |
| --- | --- | --- | --- | --- | --- | --- |
| 30 | 385 | 127 | 13 | 0.752  [0.713, 0.787] | 0.967  [0.945, 0.981] | 0.846  [0.820, 0.870] |
| 40 | 384 | 98 | 14 | 0.797  [0.758, 0.830] | 0.965  [0.942, 0.979] | 0.873  [0.848, 0.895] |
| 50 | 384 | 76 | 14 | 0.835  [0.798, 0.866] | 0.965  [0.942, 0.979] | 0.895  [0.873, 0.915] |
| 60 | 357 | 68 | 41 | 0.840  [0.802, 0.872] | 0.897  [0.863, 0.923] | 0.868  [0.842, 0.892] |
| 70 | 340 | 55 | 58 | 0.861  [0.823, 0.891] | 0.854  [0.816, 0.886] | 0.858  [0.831, 0.883] |

TP, true positive; FP, false positive; FN, false negative; PPV, positive predictive value; CI, confidence interval; YOLOv11-RSD, You Only Look Once version 11-rare sperm detection.
